# Editorial: Role of acquired brain injury in brain-aging: new insight and evidence

**DOI:** 10.3389/fnins.2024.1408921

**Published:** 2024-04-17

**Authors:** Manish Kumar, Rati Sharma

**Affiliations:** Department of Pediatrics-Pulmonary, University of Alabama at Birmingham, Birmingham, AL, United States

**Keywords:** acquired brain injury (ABI), brain-aging, senescence, Symbol Digit Modalities Test (SDMT), Guttmann Cognitest

Acquired brain injury (ABI) covers brain damage resulting from **traumatic** and **non-traumatic brain causes**. Traumatic brain injury (TBI) stems from single or multiple head impacts leading to various conditions such as concussion, skull fracture, epidural, subdural, and subarachnoid hemorrhage, and penetrating brain injury. Conversely, non-traumatic brain injury arises from internal diseases such as meningitis, encephalitis, anoxia, or brain stroke. It is worth noting that ABI typically excludes any brain injury stemming from congenital disorders, degenerative diseases, or birth-related trauma (Goldman et al., 2022).

Brain aging refers to the time-dependent decline in brain function caused primarily by the gradual accumulation of cellular damage over one's lifespan. Recent findings indicate that ABI accelerates brain aging (Gonneaud et al., 2021; Spitz et al., 2022). Nevertheless, the underlying molecular mechanisms and their interactions with various neuropathological processes remain unclear. This issue focuses on exploring the impact of ABI on brain aging.

The current Research Topic features four original research articles that delve into the intricate interplay between ABI and aging. These studies highlight recent advancements in the diagnosis and treatment of brain abnormalities and associated conditions in the elderly population.

An essential aspect of brain aging is the progressive accumulation of senescent cells, which release factors that contribute to brain atrophy and degeneration, resulting in cognitive, memory, and motor function decline. In this context, Wang et al. present the first evidence of increased senescent cells in the brain post-TBI. The authors also demonstrate that administering the senolytic drugs dasatinib and quercetin can improve long-term outcomes after TBI.

Given that current medical technologies cannot detect aged brain cells or their markers, clinicians often rely on cognitive and motor function tests to assess brain aging. Early detection of cognitive impairment is critical to mitigating brain aging in ABI patients. In their study, Cattaneo et al., validate the Guttmann Cognitest against classical neuropsychological tests in older adults and ABI patients. Their findings show that the Guttmann Cognitest is a promising digital tool for assessing cognitive function, offering correlations with traditional tests and the potential for large-scale population screening and clinical research.

Another article by Wenger et al. explores the impact of cortical and periventricular lesions on the relationship between global network metrics and information processing speed in people with multiple sclerosis (pwMS). They identify non-linear relationships between increased focal cortical and periventricular damage, network properties, and the Symbol Digit Modalities Test (SDMT).

Brain plasticity is crucial for recovery post-injury. The age-specific decline in brain plasticity affects the efficacy of therapeutic interventions such as electroacupuncture (Jia et al., 20203) and remote ischemic conditioning (RIC) (Zhao et al., 2019). Huo et al. investigate the effect of aging on the outcome of electroacupuncture treatment, revealing differential age effects on cerebral metabolic mechanisms in rats with TBI. Their study underscores the importance of developing new rehabilitation strategies for an aging TBI population.

In conclusion, this Research Topic offers novel insights into the association between aging and the pathophysiology of acquired brain injury, providing valuable clinical, epidemiological, and experimental data. We anticipate that this special edition will inspire future research to identify signaling molecules and molecular pathways as potential drug targets to promote healthy aging.

## Author contributions

MK: Writing – review & editing, Writing – original draft, Supervision, Conceptualization. RS: Writing – review & editing, Writing – original draft.
